# Supplementary material for: Complexome profiling of the Chlamydomonas psb28 mutant reveals TEF5 as an early PSII assembly factor
Source: Plant Cell. 2025 Mar 17;37(6):koaf055. doi: 10.1093/plcell/koaf055 (PMC12164590; doi:10.1093/plcell/koaf055)
Supplement: koaf055_Supplementary_Data [file koaf055_supplementary_data.zip › psb28_interactive.zip!/plots/intensity/qualityPlots/peptides/_Cre16.g674800.t1.2.html]

### Cre16.g674800.t1.2

Trivial name:   
SeqCoverage [median]: 16.90  
Mass: 25.46 kDa  
  
 

| Sequence | MissedCleavage | Mass [Da] | wtIntensity | muIntensity | Score |
| --- | --- | --- | --- | --- | --- |
| ADLPADSAK | 0 | 886.44 | 0.00 | 627.86 | 63.42 |
| KYDDAVEVTFDR | 1 | 1456.68 | 0.00 | 390.60 | 38.30 |
| LTGTALVSNVK | 0 | 1101.64 | 0.00 | 2730.90 | 125.71 |
| MSSALSTR | 0 | 851.42 | 249.32 | 585.31 | 79.71 |
|  |  |  | 249.3 | 4334.7 |  |
